# First record of *Lutzomyia cruzi* and phlebotomine sand fly diversity in a rural settlement of the metropolitan area in Central-west Brazil

**DOI:** 10.1590/S1984-29612025074

**Published:** 2026-04-27

**Authors:** Deise Cristina Macanham, James Moraes de Moura, Jaqueline Aparecida Menegatti, Cladson de Oliveira Souza, Álvaro Felipe de Lima Ruy Dias

**Affiliations:** 1 Universidade de Cuiabá – UNIC, Faculdade de Medicina Veterinária, Programa de Pós-graduação Stricto Sensu em Biociência Animal, Cuiabá, MT, Brasil; 2 Instituto Federal de Educação Ciência e Tecnologia de Mato Grosso – IFMT, Cuiabá, MT, Brasil; 3 Laboratório Central de Saúde Pública – Lacen, Secretaria de Estado de Saúde, Cuiabá, MT, Brasil

**Keywords:** Vector, zoonoses, Mato Grosso, sand fly, Vetor, zoonoses, Mato Grosso, flebotomíneos

## Abstract

The state of Mato Grosso harbors a high species richness of phlebotomine sand flies, among which *Lutzomyia longipalpis* and *Lutzomyia cruzi* stand out as recognized vectors of the protozoan *Leishmania infantum*, the etiological agent of visceral leishmaniasis. This study aimed to conduct an entomological survey in a rural settlement located in the municipality of Várzea Grande, Mato Grosso, Brazil. Collections were carried out between September 2023 and March 2024 at ten sampling points distributed across residential (RZ) and agricultural (AZ) zones. A total of 220 phlebotomine sand flies were captured, belonging to six genera and eleven species. The most abundant species was *Evandromyia lenti*. Three species of recognized epidemiological importance were identified: *Lu. cruzi, Lu. longipalpis* and *Nyssomyia whitmani*, with the latter recorded for the first time in Várzea Grande municipality. The results indicate low diversity and evenness in both sampled environments, with greater community balance in the AZ and higher dominance in the RZ. These findings suggest low diversity, high dominance, and potential ecological imbalance, particularly in areas with a higher degree of anthropogenic disturbance. The novel record of *Lu. cruzi* highlights the need for continuous entomological surveillance given the potential risk of leishmaniasis transmission in the region.

## Resumo

O estado de Mato Grosso abriga uma grande diversidade de flebotomíneos, entre os quais se destacam *Lutzomyia longipalpis* e *Lutzomyia cruzi*, ambas reconhecidamente envolvidas na transmissão do protozoário *Leishmania infantum*, agente causador da leishmaniose visceral. O presente estudo teve como objetivo realizar um levantamento entomológico em um assentamento rural localizado no município de Várzea Grande, Mato Grosso, Brasil. As coletas foram realizadas entre setembro de 2023 e março de 2024, em dez pontos distribuídos entre zonas habitacional (ZH) e agrícola (ZA). Foram capturados 220 flebotomíneos, pertencentes a 6 gêneros e 11 espécies. A espécie mais abundante foi *Evandromyia lenti*. Três espécies de reconhecida relevância epidemiológica foram identificadas: *Lu. cruzi, Lu. longipalpis* e *Nyssomyia whitmani*, sendo esta última registrada pela primeira vez no município de Várzea Grande. Os resultados indicam baixa diversidade e equitatividade em ambos os ambientes amostrados, com maior equilíbrio na ZA e maior dominância na ZH. Esses dados sugerem baixa diversidade, alta dominância e possível desequilíbrio ecológico, especialmente em ambientes com maior grau de antropização. O registro inédito de *Lu. cruzi* reforça a necessidade de vigilância entomológica contínua diante do potencial risco de transmissão das leishmanioses na região.

Visceral leishmaniasis (VL), caused by the protozoan *Leishmania infantum*, is a zoonosis with high lethality and wide distribution in the Americas, particularly in regions with socio-environmental conditions favorable to vector proliferation (PAHO, 2024). In Brazil, the disease is endemic and associated with factors such as unplanned urbanization, the presence of infected dogs, and the geographic expansion of phlebotomine sand fly vectors (Brasil, 2017).

The epidemiology of VL is complex, with transmission linked to phlebotomine sand flies of the genus *Lutzomyia*, particularly *Lutzomyia longipalpis* and *Lutzomyia cruzi* (Thies et al., 2023). In the state of Mato Grosso, entomological surveys conducted between 1996 and 2021 recorded the presence of *Lu. cruzi* in 41 municipalities and *Lu. longipalpis* in 57. In Várzea Grande, a municipality endemic for the disease, only *Lu. longipalpis* had been detected until recently (Thies et al., 2023).

This scenario is particularly concerning, as the simultaneous presence of multiple vector species in the same area may enhance the transmission of *L. infantum*, especially in rural regions characterized by intense interaction between humans, animals, and the environment. Nevertheless, significant gaps remain in the mapping of phlebotomine sand flies in municipalities of Mato Grosso classified as priorities for VL control actions (Menegatti & Dias, 2024).

Investigating and recording the diversity and distribution of these species in endemic areas are essential for understanding their behavior and supporting surveillance and control strategies, particularly in light of the geographic expansion of secondary vectors into new areas. In this context, the present study aimed to conduct an entomological survey in a rural settlement of the São Miguel Arcanjo community, located in the municipality of Várzea Grande, state of Mato Grosso, Brazil.

The municipality of Várzea Grande – MT, is located 7 km from Cuiabá, the state capital, on the right bank of the Cuiabá River. It covers an area of 938.057 km^2^ and has a population of 300.078 inhabitants (Brasil, 2023), with a tropical climate, well-defined dry and rainy seasons, and high temperatures (Brasil, 2025). The study was conducted in the rural settlement of São Miguel Arcanjo (15°29′47″S, 56°27′55″W), which comprises 70 smallholder farms dedicated to family-based agriculture and is located 45 km from the municipal center of Várzea Grande.

The entomological survey ten employed CDC (Centers for Disease Control and Prevention) light traps, one trap per point, installed in the peridomestic area of ten georeferenced properties (P1–P10, GPS–Timestamp) for three consecutive nights each month, being placed in the late afternoon and collected in the morning. Sampling points were classified according to their location: P1, P2, P3, P4, and P10 were situated within the settlement’s residential core - an area of higher population density, whereas P5, P6, P7, P8, and P9 were located in the agricultural zone, dispersed rural areas.

Four sampling campaigns were carried out between September 2023 and March 2024, with 60-day intervals to account for dipteran reproductive cycles. The insects were euthanized by freezing, sorted, and only phlebotomine sand flies were preserved in 70% ethanol, mounted on slides, and identified taxonomically (Galati, 2018). The generic names of the phlebotomine sand fly species were abbreviated according to the standardization proposed by Marcondes, (2007).

To estimate phlebotomine biodiversity in the sampled areas, several ecological indices were calculated based on species abundance per sampling point, grouped into: settlement residential zone (RZ) **-** high population density**-** and settlement agricultural zone (AZ) - dispersed rural areas. Minitab software was used to calculate the Shannon index (H′) and Simpson diversity index (1 − D). Evenness was assessed using Pielou’s index (J′). Estimated species richness (S^Chao1^) was obtained using the Chao1 estimator, which accounts for rare species. Species dominance at different sampling points was measured using the Berger–Parker index (BP). Rarefaction curves were generated for each sampling point to estimate species richness using RStudio software.

A total of 220 phlebotomine sand flies were collected, including 115 males (52.27%) and 105 females (47.72%), distributed monthly as follows: September 2023 – 38 (17.27%); November 2023 – 5 (2.27%); January 2024 – 104 (47.27%); and March 2024 – 73 (33.18%). Eleven species were identified, belonging to six genera: *Evandromyia, Lutzomyia*, *Martinsmyia*, *Nyssomyia*, *Psathyromyia* and *Sciopemyia*. The most abundant genus was *Evandromyia*, with 201 specimens (91.36%).

The sampling point with the highest number of specimens was P6, with 81 individuals (36.81%), followed by P1, with 50 individuals (22.72%) (Table 1). Points of greatest public health relevance were P6, P1, and P9, which yielded specimens of *Lu. cruzi*, *Lu. longipalpis*, and *Ny. whitmani*. The most abundant species were *Ev. lenti*, representing 77.72% (n = 171), and *E. evandroi*, representing 9.54% (n = 21); together, they accounted for 87.26% of the collected specimens. The species *Sciopemyia sordellii*, *Psathyromyia punctigeniculata*, and *Martinsmyia oliveirai* were each represented by a single specimen.

**Table 1:** Number of phlebotomine sand flies captured at different sampling points between September 2023 and March 2024 in the rural settlement of São Miguel Arcanjo, Várzea Grande, Mato Grosso, Brazil.

Phlebotomine sand fly species	P1		P2		P3		P4		P5		P6		P7		P8		P9		P10	
	♂	♀	♂	♀	♂	♀	♂	♀	♂	♀	♂	♀	♂	♀	♂	♀	♂	♀	♂	♀
***Evandromyia evandroi***	0	1	0	1	0	1	0	3	0	1	4	1	0	2	1	1	1	3	1	0
***Evandromyia lenti***	26	17	3	0	0	0	4	5	3	4	44	23	3	4	5	5	6	3	5	11
***Evandromyia sallesi***	0	3	0	0	0	0	0	0	0	0	0	0	0	0	0	0	0	0	0	0
***Evandromyia saulensis***	0	0	0	0	0	0	0	0	0	0	0	1	0	0	0	0	0	0	0	1
***Evandromyia termitophila***	0	0	1	0	0	0	0	0	0	1	0	1	1	0	0	0	0	0	0	0
***Lutzomyia cruzi***	1	0	0	0	0	0	0	0	0	0	1	0	0	0	0	0	1	0	0	0
***Lutzomyia longipalpis***	1	0	0	0	0	0	0	0	0	0	1	0	1	0	0	0	0	0	0	0
***Lutzomyia longipalpis/cruzi*** ^*^	0	0	0	0	0	0	0	0	0	0	0	4	0	0	0	0	0	0	0	0
***Martinsmyia oliverai***	0	0	1	0	0	0	0	0	0	0	0	0	0	0	0	0	0	0	0	0
***Nyssomyia whitmani***	0	1	0	0	0	0	0	0	0	0	0	1	0	1	0	0	0	1	0	0
***Psathyromyia punctigeniculata***	0	0	0	0	0	0	0	0	0	0	0	0	0	0	1	0	0	0	0	0
***Sciopemyia sordellii***	0	0	1	0	0	0	0	0	0	2	0	0	0	0	0	0	0	0	0	0
*Total by sex*	28	22	6	1	0	1	4	8	3	8	50	31	5	7	7	6	8	7	6	12
**Total**	50 (22.72%)		7 (3.18%)		1 (0.45%)		12 (5.45%)		11 (5.00%)		81 (36.81%)		12 (5.45%)		13 (5.90%)		15 (6.81%)		8 (8.18%)	

P1 to P10 – sampling points: P1, P2, P3, P4, and P10 (residential zone, RZ), P5, P6, P7, P8, and P9 (agricultural zone, AZ). *Lutzomyia longipalpis/cruzi – Females of these species are morphologically indistinguishable. For diversity index calculations, the four indistinct female specimens were proportionally redistributed between the two species based on the proportion of captured males, which was 50% for each species. Accordingly, two specimens were assigned to *Lu. longipalpis* and two to *Lu. cruzi*. ♂ – male. ♀ – female.

Several diversity indices were calculated and are presented in Table 2. In the RZ, 88 individuals were captured, distributed among ten species, whereas in the AZ, abundance was higher (132 individuals), but observed richness was slightly lower, with nine species identified. Shannon’s diversity index (H′), which increases with community diversity, was higher in the AZ (0.970), as was Simpson’s index (1 – D = 0.412), also indicating greater diversity in this environment. Nonetheless, the relatively low values of both indices reveal low diversity in both sampled zones.

**Table 2:** Diversity indices applied to the phlebotomine sand fly fauna collected at ten sampling points classified as residential zone (RZ – area of population clustering) and agricultural zone (AZ – scattered rural areas) in the rural settlement of São Miguel Arcanjo, Várzea Grande, Mato Grosso, Brazil.

**Indices**	**Residential zone (RZ)**	**Agricultural zone (AZ)**
**Total individuals**	88	132
**Observed richness (S^obs^)**	10	9
**Shannon diversity index (H’)**	0.846	0.97
**Simpson diversity (1-D)**	0.341	0.412
**Pielou’s evenness (J’)**	0.367	0.441
**Berger-Parker dominance index (BP)**	0.807	0.758
**Estimated richness (S^Chao1^)**	31	9.5

Residential zone (P1, P2, P3, P4, and P10), Agricultural zone (P5, P6, P7, P8, P9).

**Figure 1:**
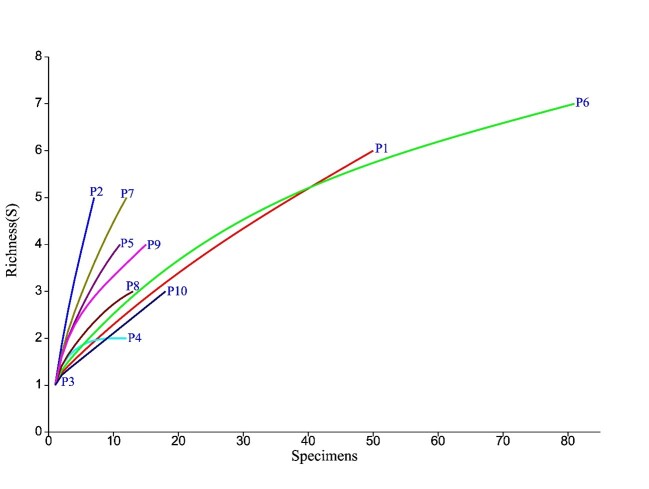
Rarefaction curves of phlebotomine species richness recorded at the sampling points (P1–P10) in the São Miguel Arcanjo settlement, Várzea Grande, Mato Grosso, Brazil. The x-axis represents the number of individuals sampled, and the y-axis indicates the observed species richness, allowing comparisons among sampling points and the evaluation of sampling effort sufficiency.

The rarefaction curves demonstrated variation in species richness among the different sampling points (P1 to P10). Sample P6 exhibited the highest observed richness and maintained an upward trend even with an increase in the number of specimens, suggesting the possibility of unsampled species. In contrast, P3 exhibited the lowest richness, indicating low diversity. Samples such as P1, P6, and P10 showed more elongated curves, reflecting more diverse or under-sampled communities, while P2, P5, P7, P8, and P9 quickly reached a plateau, suggesting lower diversity or the predominance of a few species (Figure 1).

Pielou’s evenness index (J′), which ranges from 0 (high inequality) to 1 (perfectly uniform distribution), was higher in the AZ (0.441). However, the obtained values indicate that species distribution is uneven in both environments, suggesting a stronger dominance of a single species. The Berger–Parker dominance index (BP), which ranges from 0 (minimal dominance) to 1 (total dominance by a single species), reinforces this pattern, showing high values in both RZ (0.807) and AZ (0.758).

Estimated species richness (S^Chao1^) suggested a possible underestimation of diversity in the RZ (31). This value may be related to the presence of several rare species (represented by a single individual) and the possible absence of doubletons, which inflates richness estimates under the model.

This study records, for the first time, the occurrence of *Lu. cruzi* in the municipality of Várzea Grande, Mato Grosso, expanding knowledge on the distribution of this secondary vector of visceral leishmaniasis (VL) in the Brazilian Midwest. Furthermore, due to the post-pandemic period, phlebotomine sand fly monitoring in the municipality had been interrupted, making this work the most recent record of phlebotomine sand fly fauna in Várzea Grande and the only survey conducted so far in the studied settlement area.

Although all species identified here have been previously reported in Mato Grosso (Thies et al., 2023), these findings indicate changes in abundance patterns described in the literature. In contrast to the entomological profile reported by Missawa et al., (2011), Alves et al., (2012), and Thies et al., (2023), which identified *Ny. whitmani* as the predominant species, the present study revealed the dominance of *Ev. lenti*, recorded in 90% of the sampled sites (n = 9/10), and *E. evandroi*, present in all sampled sites (n = 10/10).

The high abundance of these species may be associated with their known tolerance to peridomestic environments and preserved forest fragments, features predominant at the trap installation sites. Supporting these findings, De Castilho Sanguinette et al., (2015) reported the absolute predominance of *Ev. lenti* in wild areas and *E. evandroi* in transitional areas, highlighting the ecological plasticity of these phlebotomine sand flies.

It is worth noting the scarcity of recent studies on the habits and biology of *Ev. lenti*, which may be attributed to its assumed low medical relevance (Brazil et al., 1997). However, given its abundant presence in peridomestic and rural areas, it is essential to investigate its potential interactions with domestic reservoirs and, consequently, its possible role in the transmission cycle of zoonotic agents. Although the vector competence of *Ev. lenti* remains uncertain, its abundance and ecological behavior warrant further investigative approaches.

Although the São Miguel Arcanjo settlement comprises areas with different patterns of land occupation, with a more densely populated central nucleus and an agricultural zone consisting of more dispersed properties, both are located in rural environments and share environmental elements that favor the occurrence and maintenance of phlebotomine sand fly populations. Common features include the presence of chicken coops, stray dogs, cultivated areas, and proximity to dense vegetation, which together provide shelter, food sources, and favorable climatic conditions for the survival and proliferation of these insects (Silva et al., 2022; Souza et al., 2023).

It is noteworthy that all ten sampling sites had chicken coops in the surroundings of the traps. The literature has already recognized chicken coops as potential risk factors for the maintenance of phlebotomine sand fly populations, not only by providing shelter and organic matter for the development of immature forms, but also by attracting blood-seeking females (Souza et al., 2023). These features concentrate vectors in peridomestic areas and facilitate contact with secondary hosts, such as dogs, which act as domestic reservoirs of VL (Dantas-Torres, 2007; Vilas-Boas et al., 2024).

The simultaneous detection of *Lu. longipalpis* and *Lu. cruzi*, proven vectors of *L. infantum*, associated with the presence of *Ny. whitmani*, the main vector of *L. (Viannia) braziliensis*, outlines a complex and concerning epidemiological scenario. The coexistence of these vector species, with different vector competences and associated etiological agents, increases the risk of exposure of humans and animals to multiple transmission cycles of leishmaniases, requiring broader entomological surveillance measures and a revision of epidemiological surveillance strategies, which still predominantly focus on educational campaigns and animal control measures (Menegatti & Dias, 2024).

The data obtained in this study contrast with the findings of other research conducted in Mato Grosso (Alves et al., 2012; Thies et al., 2023; Nascimento et al., 2024), which report abundant collections of medically important phlebotomine sand flies, such as *Lu. longipalpis*, throughout the year. In our investigation, however, only three specimens of this species were recorded, distributed across sites P1, P6, and P7, reinforcing the need to consider local and seasonal factors when analyzing vector dynamics. A similar finding was reported by Campelo et al. (2014), who observed high abundance of *Lu. cruzi* and *Ny. whitmani*, but low frequency of *Lu. longipalpis*.

The diversity indices applied in this study show that, just as the observed richness (S^obs^) of phlebotomine sand flies was equal in the RZ and AZ, the ecological structure of the communities, both located in rural environments, did not present significant differences. The low values of Shannon (H’) and Simpson (1–D) indices in both environments indicate low diversity, associated with high dominance of a single species, possibly *Ev. lenti*, as evidenced by the Berger-Parker dominance index (BP). This pattern is more pronounced in the RZ, which may be related to anthropogenic features such as population density, presence of domestic animals, modified microclimates, and organic waste that favor the proliferation of synanthropic species (Chagas et al., 2018). Low diversity is an important ecological warning, as communities with low diversity and high dominance tend to increase the risk of pathogen transmission, especially when the dominant species is a competent vector.

The Shannon index (H’) reflects both species richness and evenness (J’). In the RZ, moderate richness (ten species) combined with low evenness (J’ = 0.367) resulted in lower diversity (H’ = 0.846), indicating that a few species dominate the community. In contrast, in the AZ, slightly lower richness (nine species) but higher evenness (J’ = 0.441) led to higher diversity (H’ = 0.97), suggesting a more balanced distribution of individuals among species. These patterns highlight that differences in H’ between localities are influenced not only by species richness but also by the relative abundance of species, reinforcing the importance of considering both components when interpreting diversity in phlebotomine communities.

Similarly, Pielou’s evenness index (J’) indicates low evenness in the distribution of species, that is, a clear imbalance in abundance among the species present in each environment. The AZ showed a slightly higher value, indicating greater ecological balance, possibly influenced by greater vegetation cover, lower urban intervention, and distinct microhabitats, which favor a more diverse phlebotomine sand fly fauna and reduce dominance by synanthropic or opportunistic species (Ramos et al., 2014).

The high value of the richness estimator (S^Chao1^ = 31) in the RZ can be explained by the absence of doubletons (species represented by two individuals) and the expressive number of singletons in the sampling. Since the S^Chao1^ calculation is sensitive to the ratio between these two groups, under-detection of less abundant species may have inflated the richness estimate (Feitosa et al., 2012). This pattern indicates not only the presence of rare species within the community but also a limitation of the sampling effort in capturing them with sufficient representativeness.

The results observed in this study, characterized by low diversity and strong dominance of a single species, follow the pattern described by Ramos et al., (2014) in Rio Pardo, Amazonas, where dominance of a few vector species adapted to rural settlement areas, such as *Nyssomyia antunesi* and *Nyssomyia umbratilis*, was also recorded. This convergence suggests that, regardless of total diversity, there is a trend toward synanthropization of vectors in human-occupied areas, particularly in the presence of domestic animals, organic waste, and microclimatic alterations, as observed in our study.

The use of a greater number and variety of traps contributes to the saturation of the species curve and increases the reliability of diversity estimates (Rodríguez-Rojas & Rebollar-Téllez, 2017; Arzamani et al., 2019). Evidence indicates that the type and spatial arrangement of traps significantly influence the representativeness of captures, potentially leading to under- or overestimations of species abundance and richness (Signorini et al., 2013). Furthermore, the use of attractants can increase the number of individuals collected, although it may introduce biases related to the sex, age, or behavior of the insects (Alexander, 2000). Therefore, methodological robustness and comparability between studies depend directly on the number and type of traps used, as well as the sampling effort employed.

Additionally, it is important to recognize as a limitation of the present study its cross-sectional design, consisting of a one-time entomological survey rather than a long-term investigation. Temporary surveys, although useful for identifying the composition and local diversity of phlebotomine sand fly fauna, do not allow proper assessment of seasonal fluctuations in species or continuous monitoring of risk factors associated with pathogen transmission.

In conclusion, the phlebotomine sand fly fauna present in the rural settlement of Várzea Grande, Mato Grosso, includes species of recognized epidemiological relevance, such as *Lu. cruzi*, *Lu. longipalpis*, and *Ny. whitmani.* This study reports, for the first time, the occurrence of *Lu. cruzi* in the municipality, representing an important finding in view of the risk of *Leishmania* spp. transmission in the region. The results indicate low diversity and evenness in both environments sampled, with greater species dominance in the RZ, possibly influenced by anthropogenic factors. In contrast, the AZ exhibited signs of greater ecological balance, possibly due to higher environmental heterogeneity. These findings reinforce the need for continuous entomological surveillance, especially in rural–urban transition areas, to support strategies for the prevention, control, and monitoring of leishmaniases.

## Data Availability

All data are available in the manuscript.
